# Exogenous Hydrogen Sulfide Promotes FAM134B-Mediated Endoplasmic Reticulum Autophagy and Inhibits Cell Aging by PERK–eIF2α and Improves Homocysteinemic Hypertensive-Induced Myocardial Fibrosis

**DOI:** 10.31083/RCM51893

**Published:** 2026-10-14

**Authors:** Chenlong Luo, Yaling Li, Jianghe Jiang, Yan Shi, Liangui Nie, Zhixiong Wu, Yiding Luo, Chun Chu, Jun Yang

**Affiliations:** ^1^The First Affiliated Hospital of Department of Cardiology, Hengyang Medical School, University of South China, 421001 Hengyang, Hunan, China; ^2^The Second Affiliated Hospital of Department of Pharmacy, Hengyang Medical School, University of South China, 421001 Hengyang, Hunan, China; ^3^Zhuzhou Central Hospital of Department of Cardiology, 412000 Zhuzhou, Hunan, China; ^4^Department of Clinical Medicine, Changsha Medical University, 410219 Changsha, Hunan, China; ^5^Hunan Provincial Key Laboratory of Multi-omics And Artificial Intelligence of Cardiovascular Diseases, University of South China, 421001 Hengyang, Hunan, China; ^6^Clinical Research Center for Myocardial Injury in Hunan Province, 421001 Hengyang, Hunan, China

**Keywords:** hyperhomocysteinemia, hydrogen sulfide, ER-phagy, FAM134B, myocardial remodeling, endoplasmic reticulum stress, hypertension

## Abstract

**Background:**

Hyperhomocysteinemia (HHCY) is a major cardiovascular risk factor closely associated with hypertension, myocardial fibrosis, and cardiac dysfunction; however, the underlying cellular mechanisms remain incompletely understood. Hydrogen sulfide (H_2_S) has emerged as an important cardioprotective gasotransmitter, yet the role of H_2_S in regulating endoplasmic reticulum (ER) stress and ER-phagy in HHCY-related cardiac remodeling remains unknown.

**Methods:**

Therefore, this study aimed to investigate the effects of exogenous H_2_S in a rat model of hyperhomocysteinemic hypertension and in H9c2 cells exposed to homocysteine and angiotensin II.

**Results:**

HHCY markedly increased blood pressure, exacerbated myocardial fibrosis, and promoted cardiomyocyte senescence, accompanied by activation of ER stress signaling and suppression of ER-phagy. Administration of H_2_S significantly alleviated these pathological changes, as evidenced by improved hemodynamic parameters, reduced collagen deposition, and decreased expression of senescence-associated markers. Mechanistically, H_2_S treatment attenuated activation of the Protein Kinase RNA-like Endoplasmic Reticulum Kinase (PERK)–Eukaryotic Initiation Factor 2α (eIF2α) pathway and enhanced Family with Sequence Similarity 134, Member B (FAM134B)-dependent ER-phagy, as indicated by increased LC3-II accumulation and augmented colocalization of FAM134B with LC3. Furthermore, silencing FAM134B substantially blunted the ability of H_2_S to restore ER-phagy and suppress cellular senescence, supporting a critical role for FAM134B in mediating the protective effects of H_2_S.

**Conclusions:**

These findings demonstrate that exogenous H2S mitigates HHCY-induced myocardial remodeling, in part, by dampening PERK–eIF2α-associated ER stress and promoting FAM134B-mediated ER-phagy. This study provides mechanistic insight into the cardioprotective actions of H_2_S in hyperhomocysteinemic cardiac injury and identifies ER-phagy as a potential therapeutic target.

## 1. Introduction

Hypertension is a major public health problem in the world’s aging population, and it is also one of the main causes of increased morbidity and mortality worldwide. Long-term pressure overload can result in cardiomyocyte hypertrophy, apoptosis, and formation of myocardial fibrosis, and eventually induce myocardial remodeling and heart failure ‎[1]. Hypertension-associated cardiac remodeling involves interactions among mechanical stress, neurohumoral stimulation, and inflammatory responses [2]. A large number of surveys have found that nearly 75% of patients with hypertension in China have Hyperhomocysteinemia (HHCY ≥10 μmol/L). Essential hypertension with elevated homocysteine is often called “H-type hypertension”. H-type hypertension is associated with an increased risk of cardiovascular target-organ damage and adverse cardiac remodeling compared with hypertension alone‎[3]. Hypertension is an age-related disease, and when combined with homocysteine levels higher than 11.84 μmol/L, the risk of all-cause death is significantly increased. Several studies have also shown that homocysteine (HCY) can induce cell senescence by activating galactosidase activity ‎[4,5]. Annoni et al. [6] also found that the expression of myocardial collagen type Ⅰ and collagen type Ⅲ was significantly increased in aging rats. Kwak et al. [7] found, *in vivo* and *in vitro*, that reactive oxygen species (ROS) production was significantly increased in aged animals and contributed to cardiac fibrosis, which further confirmed that cellular senescence is involved in the presence and development of myocardial fibrosis. Hyperhomocysteinemia contributes to cardiovascular remodeling through oxidative stress and endothelial dysfunction, and may interact with neurohumoral systems such as the renin–angiotensin system [8,9]. Therefore, inhibition of cardiomyocyte senescence is expected to become a new therapeutic target for myocardial fibrosis induced by hyperhomocysteinemia-hypertension.

Endoplasmic reticulum (ER) autophagy (ER-phagy) is a process that selectively regulates the fragmentation of the ER and is the key to ER quality control. The ER autophagy receptor FAM134B has an LC3-interacting region (LIR) at the carboxyl terminus within its cytoplasm that is able to anchor LC3 or GABA Receptor Associated Protein (GABARAP) on autophagic vesicles ‎[10]. Subsequently, ER fragments with LIR are encapsulated by autophagosomes and then sent to lysosomes for degradation ‎[11,12]. This indicates that FAM134B is a key factor mediating ER autophagy. Previous studies have shown that ER autophagy can inhibit ER stress-induced apoptosis by inducing an autophagic response ‎[13], suggesting that FAM134B-mediated ER autophagy can protect the body from ER stress-induced damage. The Protein Kinase RNA-like Endoplasmic Reticulum Kinase (PERK)/Eukaryotic Initiation Factor 2α (eIF2α) signaling pathway is one of the classic signaling pathways involved in endoplasmic reticulum stress. Activated PERK can phosphorylate the translation initiation factor eIF2α, stop protein synthesis, reduce ER load by reducing the aggregation of unfolded or misfolded proteins, and inhibit the synthesis of new proteins ‎[14]. He et al. [15] demonstrated that the PERK/eIF2α signaling pathway is involved in the occurrence and development of myocardial fibrosis. ‎ However, whether PERK/eIF2α signaling is involved in homocysteinemic hypertension-induced myocardial fibrosis through FAM134B-mediated ER autophagy remains unclear.

Hydrogen sulfide (H_2_S) is the third endogenous gas signaling molecule discovered in recent years. It is mainly produced by the endogenous enzymes cystathionine-γ-lyase (CSE), cystathionine-β-synthase (CBS), and 3-mercaptopyruvate sulfur transferase, along with cysteine aminotransferase (3-MST/CAT). It has been shown that it has anti-oxidation, autophagy-regulating, and anti-apoptotic effects. Recently, it has also been found to inhibit endothelial cell and vascular senescence ‎[16]. These protective effects are supported by recent studies demonstrating that H_2_S exerts broad cardioprotective actions, including anti-oxidative, anti-inflammatory, and anti-fibrotic effects in cardiovascular diseases [17]. Previous studies from our group have also found that hydrogen sulfide can improve myocardial fibrosis and cardiomyocyte senescence induced by diabetes, but the specific mechanism is not clear.

The precise mechanisms underlying hyperhomocysteinemia-induced myocardial remodeling remain incompletely understood, particularly regarding the interplay between ER stress, ER-phagy, and cardiomyocyte senescence. Based on previous findings, we hypothesized that hyperhomocysteinemia-induced ER stress promotes cardiomyocyte senescence and myocardial fibrosis, and that exogenous H_2_S alleviates these effects by inhibiting the PERK–eIF2α pathway and restoring FAM134B-mediated ER-phagy. Therefore, the present study aimed to investigate the role of ER stress and ER-phagy in HHCY-induced myocardial remodeling and to explore the underlying mechanisms by which H_2_S confers cardioprotection.

## 2. Materials and Methods

### 2.1 Materials

DL-homocysteine (Cat. No. H4628) and angiotensin II (AngII, Cat. No. A9525) were purchased from Sigma-Aldrich (St. Louis, MO, USA). Sodium hydrosulfide (NaHS, Aladdin, Cat. No.S106641) was purchased from Aladdin Biochemical Technology Co., Ltd. (Shanghai, China). The PERK agonist CCT020312 was obtained from MedChemExpress (MCE, Monmouth Junction, NJ, USA, Cat. No. HY-119240). Lipofectamine 3000 transfection reagent (Cat. No. L3000015) and TRIzol reagent (Cat. No. 15596026) were purchased from Invitrogen (Carlsbad, CA, USA). Primary antibodies against PERK (1:1000) (Cat. No. 20582-1-AP), eIF2α (1:1000) (Cat. No. 11170-1-AP), p-PERK (1:1000) (Cat. No. 29546-1-AP), p-eIF2α (1:1000) (Cat. No. 28740-1-AP), FAM134B (1:1000) (Cat. No. 21537-1-AP), LC3B (1:1000) (Cat. No.18725-1-AP), P16 (1:1000) (Cat. No. 10883-1-AP), P21 (1:1000) (Cat. No. 10355-1-AP), P53 (1:1000) (Cat. No. 10442-1-AP), Sirtuin 1 (SIRT1,1:1000) (Cat. No. 13161-1-AP), and Glyceraldehyde-3-Phosphate Dehydrogenase (GAPDH, 1:5000) (Cat. No. 10494-1-AP), HRP-conjugated Goat Anti-Mouse IgG (1:5000) (Cat. No. SA00001-1) and HRP-conjugated Goat Anti-Rabbit IgG (1:5000) (Cat. No. SA00001-2) were purchased from Proteintech Group, Inc. (Wuhan, Hubei, China). Other reagents used in this study were of analytical grade.

### 2.2 Cell Culture

H9c2 rat cardiomyoblasts were obtained from the Cell Bank of the Chinese Academy of Sciences (Shanghai, China). According to the supplier’s quality control documentation, species authentication was performed using PCR with species-specific primers targeting the cytochrome c oxidase subunit I (COI) gene, confirming rat origin with no detectable contamination from the other species tested. The supplier’s documentation also reported a negative mycoplasma test result. H9c2 cells were cultured in high-glucose Dulbecco’s modified Eagle’s medium (DMEM; Procell, Wuhan, Hubei, China; Cat. No. PM150210) containing 4.5 g/L glucose, 584 mg/L L-glutamine, and 110 mg/L sodium pyruvate, supplemented with 10% fetal bovine serum (FBS; Procell; Cat. No. 164210) and 1% penicillin–streptomycin solution (100×; MeilunBio; Cat. No. MA0110), yielding final concentrations of 100 U/mL penicillin and 100 μg/mL streptomycin. Cells were maintained at 37 °C in a humidified atmosphere containing 5% CO_2_. When the cells reached 1 × 10^6^, H9c2 cells were treated with 1 mM DL-homocysteine and 200 nM AngII for 24 h to induce cellular senescence [18]. The exogenous H_2_S donor 100 μM (Sodium hydrosulfide, NaHS, 100 μM) was added 30 min prior to stimulation‎. To confirm the function of H_2_S through activation of PERK/eIF2α signaling, the PERK agonist (CCT020312, 10 µM, obtained from MCE) was added half an hour after pretreatment with Sodium hydrosulfide (100 µM). They were then treated with DL-homocysteine (1 mM) and AngII (200 nM) for 24 h at 37 °C. (For HG treatments, cells were incubated with DMEM (Wuhan Pricella Biotechnology Co., Ltd., Wuhan, Hubei, China; Cat. No. PM150210) containing 33 mM glucose).

### 2.3 β-galactosidase (SA-β-Gal) Staining Was Performed

The degree of cell senescence was determined by the green fluorescent indicator β-galactosidase (SA-β-Gal). Cells in six-well plates were incubated for 24 hours under different treatments, removed, washed three times with phosphate-buffered saline (PBS, Procell, Wuhan, Hubei, China; Cat. No. PB180327), and then fixed with 4% formaldehyde (EMD Millipore, Billerica, MA, USA) for 10 minutes at room temperature before washing with PBS. SPiDER-βGal (Dojindo, Kumamoto, Japan; Cat. No. SG02-10) was added to the cells and co-incubated at 37 °C for 30 min. Finally, the cells were observed using a fluorescence microscope (OLYMPUS Corporation, Japan), and the degree of cell senescence was assessed according to the intensity of the green fluorescence ‎[19].

### 2.4 Cell Interference Technology (siRNA Transfection)

siRNA targeting FAM134B (Gene Pharma, Shanghai, China) was transiently transfected into H9c2 cells with Lipofectamine 3000 transfection reagent (Thermo Fisher Scientific, Waltham, MA, USA) according to the manufacturer’s instructions. The siRNAs were sequenced as follows:

    FAM134BsiRNA:

    si-r-Fam134b-Rat-001: GAGAGTGTATCATCTCATT

    si-r-Fam134b-Rat-002: CCACAAGGATGACAGTGAA

    si-r-Fam134b-Rat-003: GCCGCAAAGCACAGATAAA

### 2.5 Western Blotting

Cell WB: H9c2 cells were removed, and buffer containing phenylmethylsulfonyl fluoride (PMSF) (9:1) was used for radioimmunoprecipitation lysis buffer (RIPA lysis buffer) to extract total protein. Protein content was determined using the bicinchoninic acid (BCA) Protein Assay kit. RIPA lysis buffer (P0013), phenylmethylsulfonyl fluoride (PMSF, ST506), and the BCA Protein Assay Kit (P0012S) were purchased from Beyotime Biotechnology (Shanghai, China). The extracted samples were subjected to loading electrophoresis and transferred to polyvinylidene difluoride (PVDF) membranes (Millipore, Billerica, MA, USA, Cat. No. IPVH00010). The membranes were blocked with Tris-buffered saline containing 5% milk and 0.1% Tween 20 for 2 h at room temperature, incubated with the primary antibodies overnight at 4 °C, and then incubated with the appropriate horseradish peroxidase-conjugated anti-rabbit or anti-mouse IgG secondary antibodies for 1 h at room temperature. Finally, the results were collected by a Tannon 5200 workstation and analyzed by Alphalmager TM 2200 (Alpha Innotech Corp., San Leandro, CA, USA) and GraphPad Prism 5 (GraphPad Software, Inc., San Diego, CA, USA) image analysis software.

For tissue WB, cell lysate, and PMSF were added to the myocardial tissue of each group after grinding, and the supernatant was extracted and quantified by BCA colorimetry. The protein was denatured by heating to 95 ℃ for 10 min, followed by electrophoresis, membrane transfer, incubation with primary antibody overnight, and incubation with horseradish peroxidase-conjugated anti-rabbit or anti-mouse IgG as a secondary antibody for 1 hour. Grayscale analysis was also performed using image analysis software (AlphaImager TM 2200 and GraphPad Prism 5).

### 2.6 Animal Model

Fourteen male spontaneously hypertensive rats (SHR) and 14 adult male normotensive rats (WKY) were purchased from Beijing Weitong Lihua Laboratory Animal Technology Co., Ltd., weighing (200 ± 20) g. They were kept in a room at 24 ± 3 ℃, with free access to food and water, and day and night alternating every 12 hours in accordance with the standards of the Regulations for the Administration of Laboratory Animals issued by the State Science and Technology Commission of the People’s Republic of China.

After adaptive feeding for 1 week, 14 SHR received drinking water containing 1% L-methionine (10 g/L) and were randomly divided into the Model and Model+H_2_S groups (n = 7 per group). 14 WKY rats received regular drinking water and were randomly divided into the Control and Control+H_2_S groups (n = 7 per group). After 10 weeks, the blood HCY concentration of the rats was measured according to the instructions of the reagent manufacturer. In addition, the rats in the Model+H_2_S group and Control+H_2_S group were intraperitoneally injected with NaHS (56 μmol/kg/day, intraperitoneal), and the rats in the Control group and Model group were intraperitoneally injected with the same dose of normal saline for 4 weeks. WKY rats were used as normotensive controls, while SHR rats represented a genetically hypertensive background. This pairing is a well-established model for studying hypertension and its complications [20,21]. This design allows differentiation between the effects of hyperhomocysteinemia alone and hyperhomocysteinemia combined with hypertension.

At the end of the experiment, rats were anesthetized with pentobarbital sodium (50 mg/kg, intraperitoneal injection) and sacrificed for tissue collection. Euthanasia was performed by overdose of pentobarbital sodium (150 mg/kg, intraperitoneal injection) in accordance with the institutional guidelines for animal care and use. All animal experiments were approved by the Institutional Animal Care and Use Committee of University of South China (Approval Number: 11000384).

### 2.7 Reverse Transcription Quantitative PCR (RT-qPCR)

The mRNA expression levels of *Col1a2 *and *Col3a1 *in myocardial tissue were quantified by reverse transcription quantitative PCR (RT-qPCR). Trizol reagent (Invitrogen, Carlsbad, CA, USA) was used to extract total RNA from myocardial tissue, and the concentration was determined by an ultraviolet spectrophotometer (Agilent Technologies, Santa Clara, CA, USA). Reverse transcription reactions were performed using a reverse transcription polymerase chain reaction kit (Thermo Fisher Scientific, formerly Fermentas, Vilnius, Lithuania, Cat. No. K1621) with RT primers (GenScript USA Inc., Nanjing, Jiangsu, China). The mRNA expression levels of Col1a2 and Col3a1 were quantified using SYBR Green-based qPCR, with GAPDH serving as the internal reference gene. Relative expression levels were calculated using the 2^⁻ΔΔCt^ method, with the Control group as the calibrator.

### 2.8 Masson Staining

The myocardial tissue of rats was fixed by formaldehyde infiltration, embedded in paraffin, then dehydrated and cut into sections (4 μm). Masson staining was used to observe the accumulation of collagen fibers in myocardial tissue under a light microscope.

### 2.9 Electron Microscopy

The myocardial tissue was fixed with 2.5% glutaraldehyde (Sinopdrug Group Chemical Reagent Co., Ltd.). They were then sequentially fixed with 1% osmium tetroxide, rinsed with phosphoric acid irrigating solution, dehydrated with different concentrations of acetone, and solidified into 50- to 100-nm-thick sections. Ultrathin sections were observed under a transmission electron microscope (TEM; Hitachi High-Tech Corporation, Tokyo, Japan) after staining with 3% uranyl acetate and lead nitrate.

### 2.10 Statistical Analysis

Statistical analysis was performed using one-way analysis of variance (ANOVA) followed by Tukey’s multiple-comparisons test for all pairwise comparisons among groups using GraphPad Prism software (San Diego, CA, USA). Only comparisons relevant to the experimental questions are displayed in the figures, with the corresponding comparison groups specified in each figure legend. Statistical significance was determined using multiplicity-adjusted *p*-values.

## 3. Results

### 3.1 Blood Pressure and Homocysteine Levels

The blood pressure of rats was measured by the intelligent non-invasive blood pressure meter BP-2010A (purchased from Beijing Ruilong Technology Co., Ltd.), and the homocysteine content in the blood from the tail vein of rats (about 2 mL) was measured by the homocysteine determination kit (cyclic enzymatic method) (purchased from Weirikang Biotechnology Co., Ltd., Wenzhou, Zhejiang, China). Compared with the Control group, the blood pressure and HCY levels in the HHCY group and HHCY+H2S group were abnormally increased, and the difference was statistically significant (*p* < 0.05). There was no significant difference in levels between the H2S group and the Control group (*p* > 0.05), as shown in Table 1.

**Table 1.** **Expression of SBP, DBP, and Hcy in rats in each group**.

Groups	Number	SBP (mmHg)	DBP (mmHg)	HCY (μmol/L)
Control	7	135.94 ± 9.84	107.79 ± 12.23	6.6 ± 2.53
HHCY	7	245.11 ± 27.55*	170.93 ± 24.79*	17.3 ± 5.31*
HHCY+H_2_S	7	237.79 ± 25.32*	175.17 ± 19.07*	18.3 ± 4.96*
H_2_S	7	141.71 ± 6.16	115.07 ± 9.03	6.71 ± 2.0

Notes: *p < 0.05 vs Control group; Values are expressed as mean ± SEM. SBP, systolic pressure; DBP, diastolic pressure; HHCY, hyperhomocysteinemia; H_2_S, hydrogen sulfide; SEM, standard error of the mean.

### 3.2 Hydrogen Sulfide May Improve Cardiac Function in Rats With Homocysteinemic Hypertension-Induced Myocardial Fibrosis

Compared with the Control group, the heart weight/body weight (HW/BW) ratio was significantly increased and left ventricular fractional shortening (LVFS) in the HHCY group was significantly decreased in the HHCY group (*p* < 0.05). These changes were significantly reversed after H_2_S intervention (*p* < 0.05). There was no significant difference in HW/BW, left ventricular end-diastolic (LVED), left ventricular end-systolic diameter (LVESD), and LVFS between the H_2_S group and the Control group (*p* > 0.05). See (Fig. 1). These findings suggest that H_2_S improves cardiac function in homocysteinemic hypertensive rats.

**Fig. 1.**
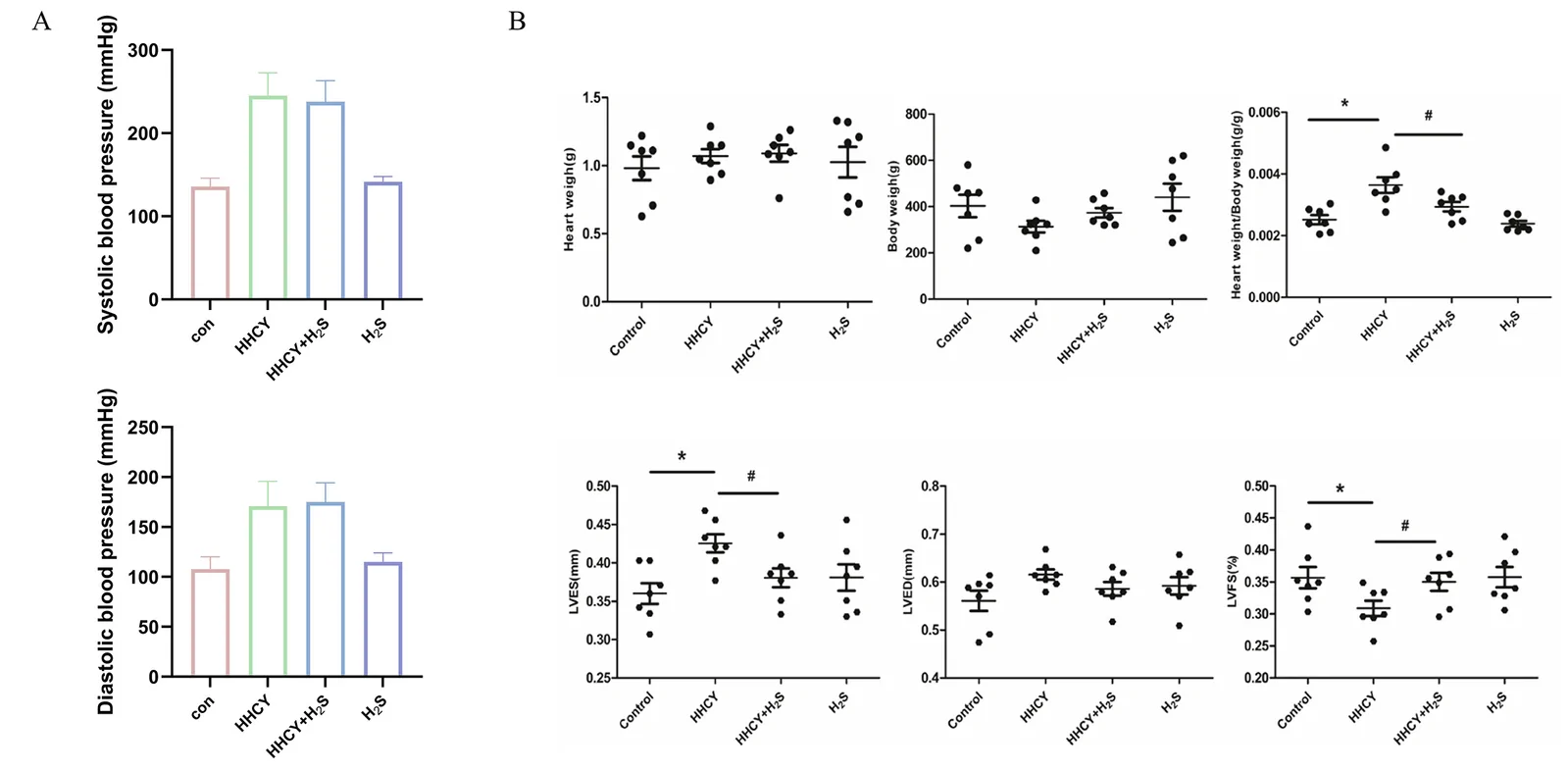
**H_2_S improves hemodynamic and cardiac structural parameters in homocysteinemic hypertensive rats**. (A) SBP and DBP measurements in the indicated groups. (B) Heart weight (HW), body weight (BW), and the heart weight-to-body weight ratio (HW/BW), left ventricular end-diastolic diameter (LVEDd), left ventricular end-systolic diameter (LVESd), and left ventricular fractional shortening (LVFS). Data are presented as mean ± SEM (n = 7–8). *p < 0.05 vs. WKY-Control group; #*p* < 0.05 vs. SHR-HHCY (Model) group.

### 3.3 Hydrogen Sulfide May Improve Homocysteinemic Hypertension-Induced Myocardial Fibrosis

Assessment of homocysteinemic hypertension-induced myocardial fibrosis using Masson staining showed that: Compared with controls, myocardial collagen fibers were significantly increased by homocysteinemic hypertension and decreased by H_2_S treatment (Fig. 2A). This was further confirmed by Western Blotting measurements of myocardial fibrin α-SMA and collagen III (Fig. 2B). These results suggest that homocysteinemic hypertension can significantly increase myocardial fibrosis, which can be reversed by hydrogen sulfide. These findings indicate that H_2_S alleviates myocardial fibrosis.

**Fig. 2.**
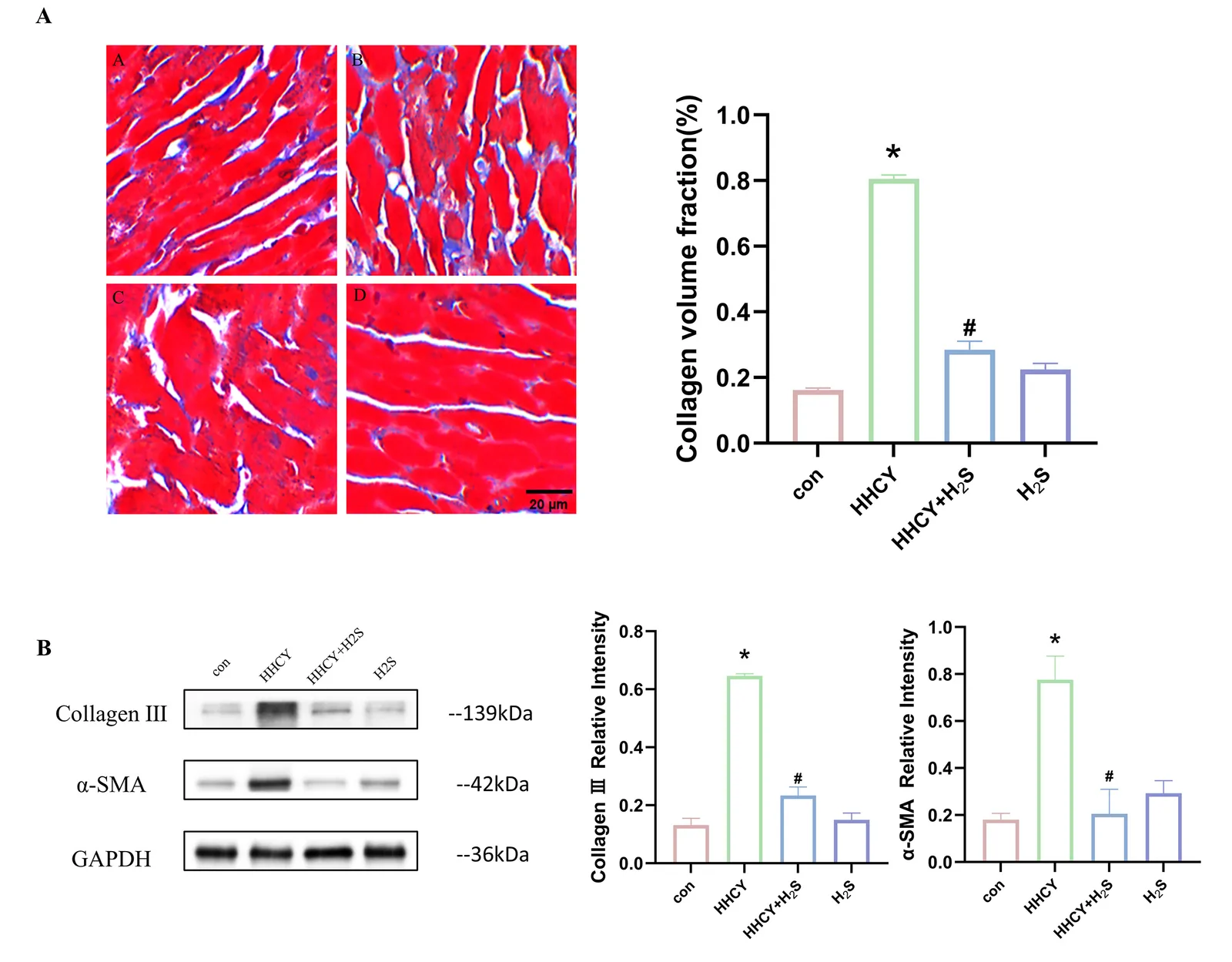
**H_2_S alleviates myocardial fibrosis in homocysteinemic hypertensive rats**. (A) Representative images of Masson’s trichrome-stained myocardial sections (collagen fibers in blue). Scale bar = 20 μm. (B) Western blot analysis (left) and quantification (right) of α-SMA and Collagen III protein levels in myocardial tissues. GAPDH served as a loading control. Data in A and B are presented as mean ± SEM (A: n = 5–6 fields per group; B: n = 3 independent experiments). *p < 0.001 vs. WKY-Control group; #*p* < 0.001 vs. SHR-HHCY (Model) group.

### 3.4 Hydrogen Sulfide May Ameliorate Homocysteinemic Hypertensive-Induced Cardiomyocyte Senescence

In our study, we found that compared with the Control group, the expression of senescence protein P16 was significantly up-regulated in the HCY group. The senescence-inhibiting protein SIRT1 was significantly down-regulated. The expression of senescence protein P16 was significantly down-regulated, and the inhibitory protein SIRT1 was significantly up-regulated after H_2_S intervention. However, compared with the control group, there was no significant difference in these proteins in the H_2_S group (Fig. 3A).

**Fig. 3.**
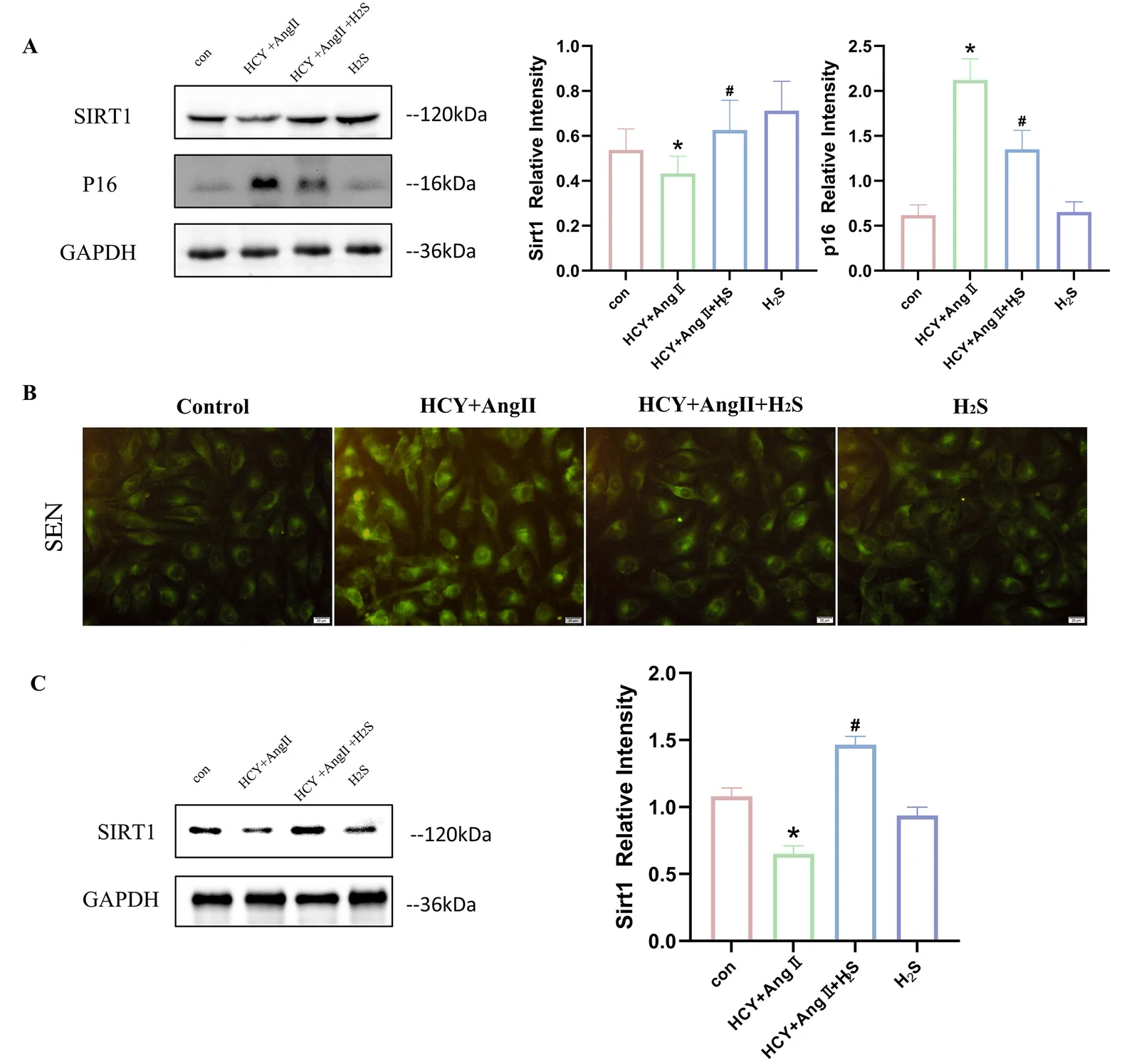
**H_2_S inhibits cardiomyocyte senescence induced by homocysteinemic hypertension**. (A) Western blot analysis and quantification of the senescence-related protein P16 and the anti-senescence-related protein SIRT1 in H9c2 cells. (B) Representative images of SA-β-Gal staining (senescent cells in green). Scale bar = 20 μm; (C) Western blot and quantification of SIRT1, GAPDH was used as a loading control. Quantitative data are the mean ± SEM from 3 independent experiments. *p < 0.05 vs. Control; #*p* < 0.05 vs. HCY+AngII group.

Cardiomyocyte senescence is involved in the development and progression of myocardial fibrosis. Therefore, we used DL-homocysteine and AngII to construct an H9c2 cardiomyocyte senescence model. First, we detected the expression of the senescence protein SIRT1 by Western blotting in the Control, HCY+AngII, HCY+AngII+H_2_S, and H_2_S groups. We found that HCY+AngII significantly reduced the expression of SIRT1, which was significantly improved by the use of hydrogen sulfide (Fig. 3A). These results indicated that the HCY+AngII group had more senescent cells. The senescence kit SPiDER-βGal (purchased from Dojindo, Japan) staining was used to observe the senescence of H9c2 cells in Control, HCY+AngⅡ, HCY+AngⅡ+H_2_S, and H_2_S. The results showed that compared with the control group, the senescence of H9c2 cells in the HCY+AngII group was significantly increased, and that this was improved with hydrogen sulfide (Fig. 3B). We used Western blot to detect the inhibitory senescence protein SIRT1 in the above 4 groups (Fig. 3C). The results showed that hydrogen sulfide intervention could inhibit the senescence of HCY+AngII cells.

### 3.5 H_2_S Can Inhibit Myocardial Cell Senescence and Homocysteinemic Hypertension-Induced Myocardial Fibrosis Through the PERK–eIF2α Pathway

Excessive endoplasmic reticulum stress often leads to the occurrence of myocardial fibrosis. The expression of PERK in the endoplasmic reticulum stress pathway was also upregulated (Fig. 4), which was significantly downregulated after H_2_S treatment. This suggests that the mechanism by which hydrogen sulfide improves homocysteinemic hypertension-induced myocardial fibrosis may be related to the endoplasmic reticulum stress pathway PERK–eIF2α.

**Fig. 4.**
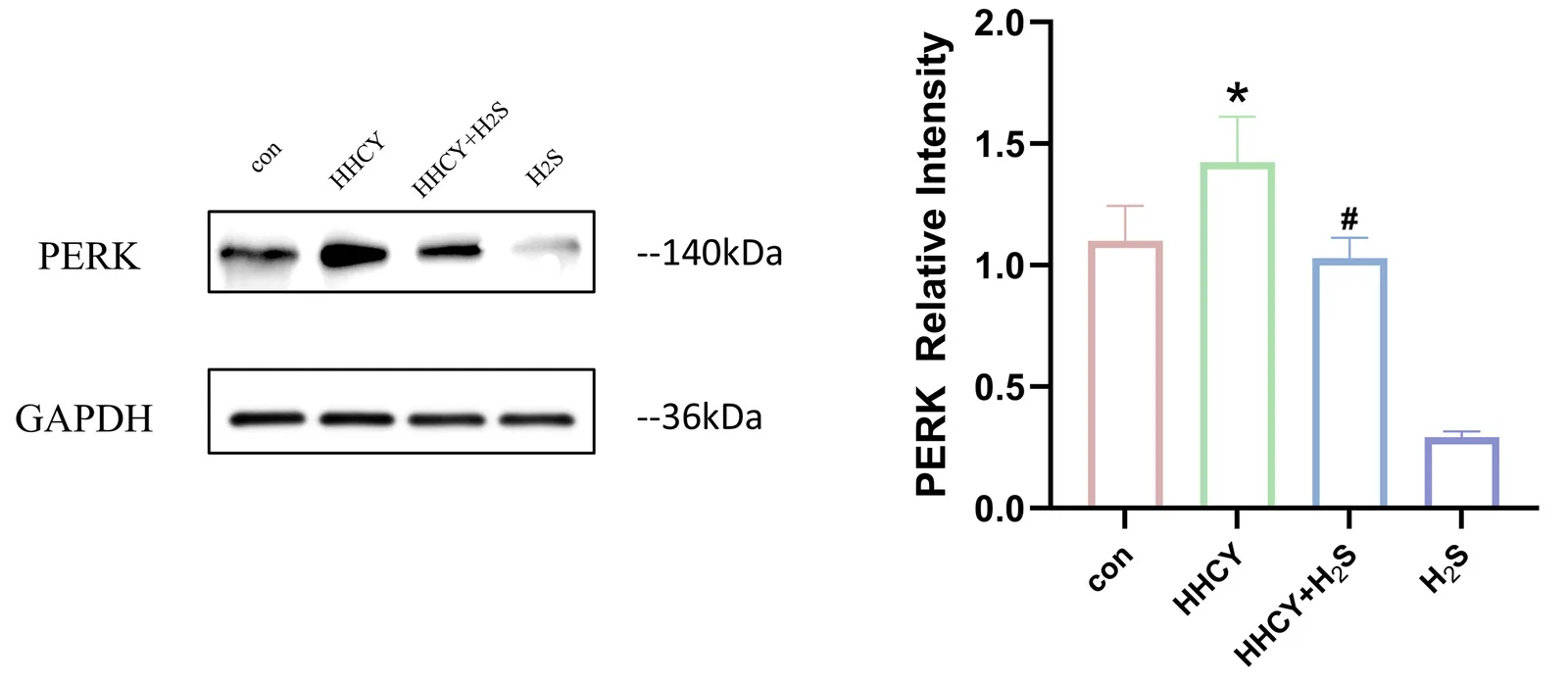
**H_2_S attenuates endoplasmic reticulum stress in the myocardium of homocysteinemic hypertensive rats**. Western blot and quantification of PERK and GAPDH were used as a loading control. Data are the mean ± SEM from 3 independent experiments. *p < 0.05 vs. WKY-Control group; #*p* < 0.05 vs. SHR-HHCY (Model) group.

To further confirm the relationship between the PERK–eIF2α signaling pathway and myocardial fibrosis, we also performed *in vitro* experiments. It has been suggested that excessive ER stress and its chronic inflammation may lead to cellular senescence ‎[17]. Therefore, Western Blotting was also used to detect the expression levels of ER stress proteins in H9c2 cardiomyocytes during senescence. When a PERK agonist was added to the culture medium, the inhibitory effect of H_2_S on cell senescence was significantly attenuated, as indicated by the increased expression of P21 (Fig. 5A). The results showed that the PERK–eIF2α pathway was significantly activated in H9c2 cells during senescence, whereas H_2_S treatment attenuated this activation (Fig. 5B). These results suggest that H_2_S can inhibit cardiomyocyte senescence through the PERK–eIF2α pathway and further improve homocysteinemic hypertension-induced myocardial fibrosis.

**Fig. 5.**
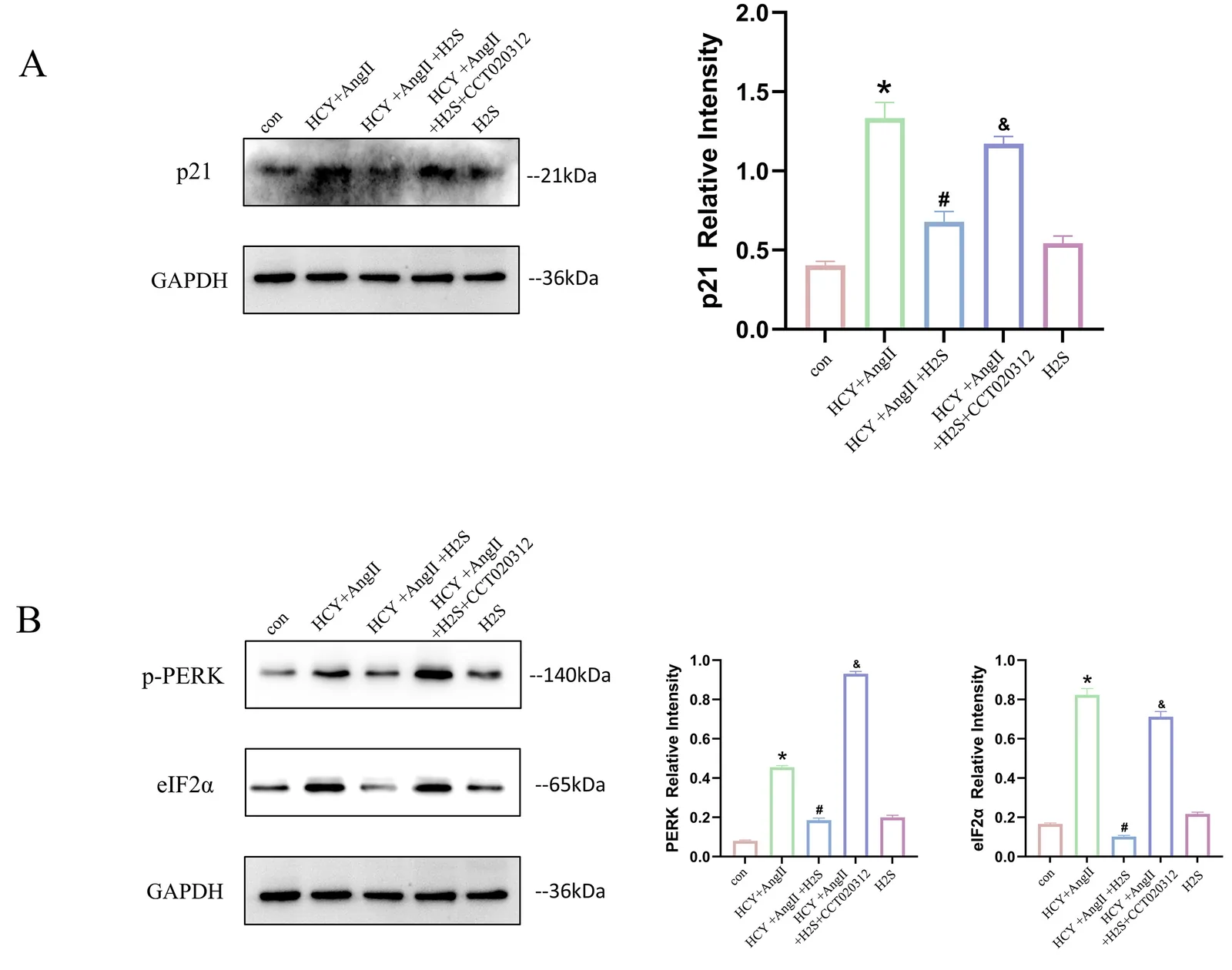
**H_2_S inhibits HCY+AngII-induced cardiomyocyte senescence via the PERK–eIF2α pathway**. (A) Western blot analysis and quantification of the senescence marker P21 in H9c2 cells. (B) Western blot analysis and quantification of eIF2α and p-PERK in cells treated with or without the PERK agonist. GAPDH was used as a loading control. Bar graphs show relative protein levels or the p-PERK ratio. Data are the mean ± SEM from 3 independent experiments. *p < 0.05 vs. Control group; #*p* < 0.05 vs. HCY+AngII group; &*p* < 0.05 vs. HCY+AngII+H_2_S group.

### 3.6 H_2_S Can Up-Regulate the Expression of FAM134B in H9c2 Cells and Myocardial Fibrosis in Rats With Hypertension and Hyperhomocysteinemia

Studies have provided conclusive evidence that FAM134B, an ER phagocytosis receptor (also known as reticular regulatory factor 1 or RETREG1), is required for mediating ER phagocytosis ‎[22]. So, does hydrogen sulfide regulate FAM134B, thereby protecting the body from injury? Western Blotting showed that H_2_S significantly increased the expression of FAM134B and LC3B both in H9c2 cells and in rats with myocardial fibrosis (Fig. 6A,B). To further verify the effect of H_2_S on FAM134B-mediated ER autophagy, we examined the occurrence of ER autophagy in H9c2 cells under fluorescence microscopy. In H9c2 cells transfected with GFP-LC3B, we found that compared with the Control group, the co-localization of FAM134B and LC3B was decreased in the HCY group, while compared with the HCY group, the co-localization of FAM134B and LC3B was significantly increased by H_2_S (Fig. 6C). These data suggest that H_2_S regulates FAM134B-mediated ER autophagy in H9c2 cardiomyocytes with hypertension and hyperhomocysteinemia and in rats with myocardial fibrosis.

**Fig. 6.**
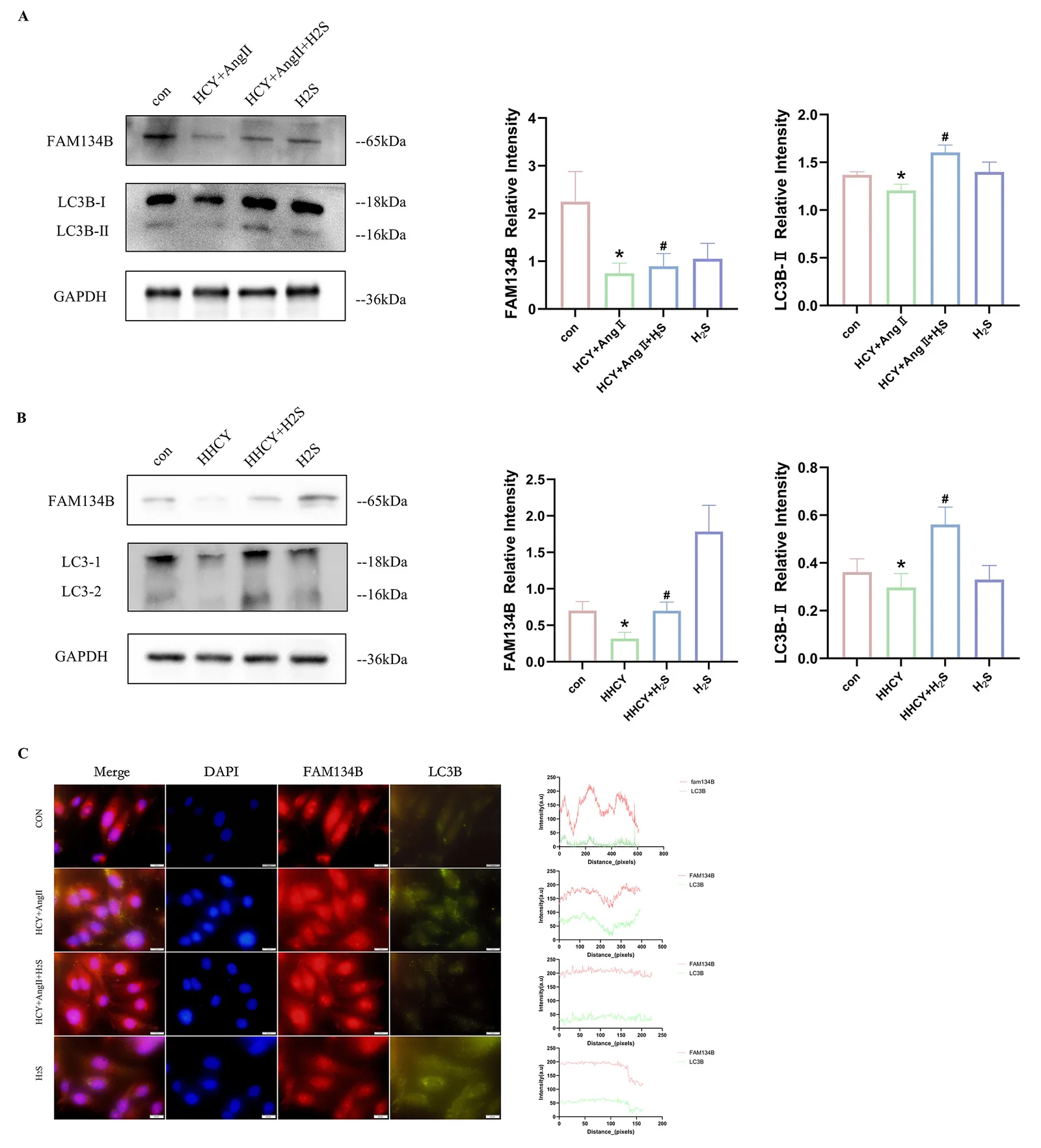
**H_2_S enhances FAM134B-mediated ER-phagy in H9c2 Cells under HCY+AngII stress**. (A) Western blot analysis of ER-phagy-related proteins (FAM134B, LC3-I/II). (B) Western blot analysis and quantitative analysis of FAM134B protein levels and the LC3-I/II ratio. (C) Immunofluorescence analysis of FAM134B (red) and LC3B (green) colocalization. Nuclei are stained with DAPI (blue). Scale bars: main panel = 20 μm. ER-phagy–related signal overlap was assessed by intensity line-scan analysis. For all Western blot panels, GAPDH was used as a loading control. Quantitative data are mean ± SEM (B: n = 3 independent experiments; n ≥ 15 cells per group from 3 experiments). *p < 0.05 vs. Control group; #*p* < 0.05 vs. HCY+AngII group.

### 3.7 H_2_S Plays a Protective Role by Inhibiting the PERK–eIF2α Pathway and Triggering FAM134B-Mediated Endoplasmic Reticulum Autophagy in H9c2 Cardiomyocytes

The above studies have confirmed that HCY+AngⅡ can activate the ER stress pathway PERK–eIF2α, while FAM134B-mediated autophagy is weakened in H9c2 cells. Therefore, in order to further explore the relationship between H2S and the ER stress pathway PERK–eIF2α and FAM134B-mediated ER autophagy, Western Blotting showed that PERK and eIF2α were significantly upregulated in the HCY+AngⅡ group compared with the Control group. FAM134B was significantly down-regulated. After adding hydrogen sulfide, the above phenomena were reversed. The results showed that, compared with the H2S+HCY+AngⅡ group, in the H_2_S+HCY+AngⅡ+CCT020312 group, FAM134B, and LC3A/B were significantly down-regulated. This demonstrates that H_2_S can upregulate FAM134B-mediated ER autophagy in H9c2 cardiomyocytes by inhibiting the PERK–eIF2α pathway and has a protective role in the human body.

### 3.8 SiRNA-FAM134B Can Antagonize Hydrogen Sulfide to Inhibit Cardiomyocyte Senescence and Improve Homocysteinemic Hypertensive-Induced Myocardial Fibrosis

We sought to further clarify whether H_2_S ameliorates homocysteinemic hypertensive-induced myocardial fibrosis through the PERK–eIF2α pathway and whether this is related to FAM134B-mediated endoplasmic reticulum autophagy. We designed three FAM134B siRNA groups (FAM134B-RAT-001; FAM134B-Rat-002; FAM134B-Rat-003). We found that FAM134B-Rat-003 was the most effective strand for interference. The expression of cell senescence proteins P53 and P21 was significantly increased in HCY+AngII+H_2_S+Si-FAM134B and Si-FAM134B groups (Fig. 7). These results suggest that FAM134B-mediated ER autophagy is essential for the inhibition of senescence in H9c2 cells.

**Fig. 7.**
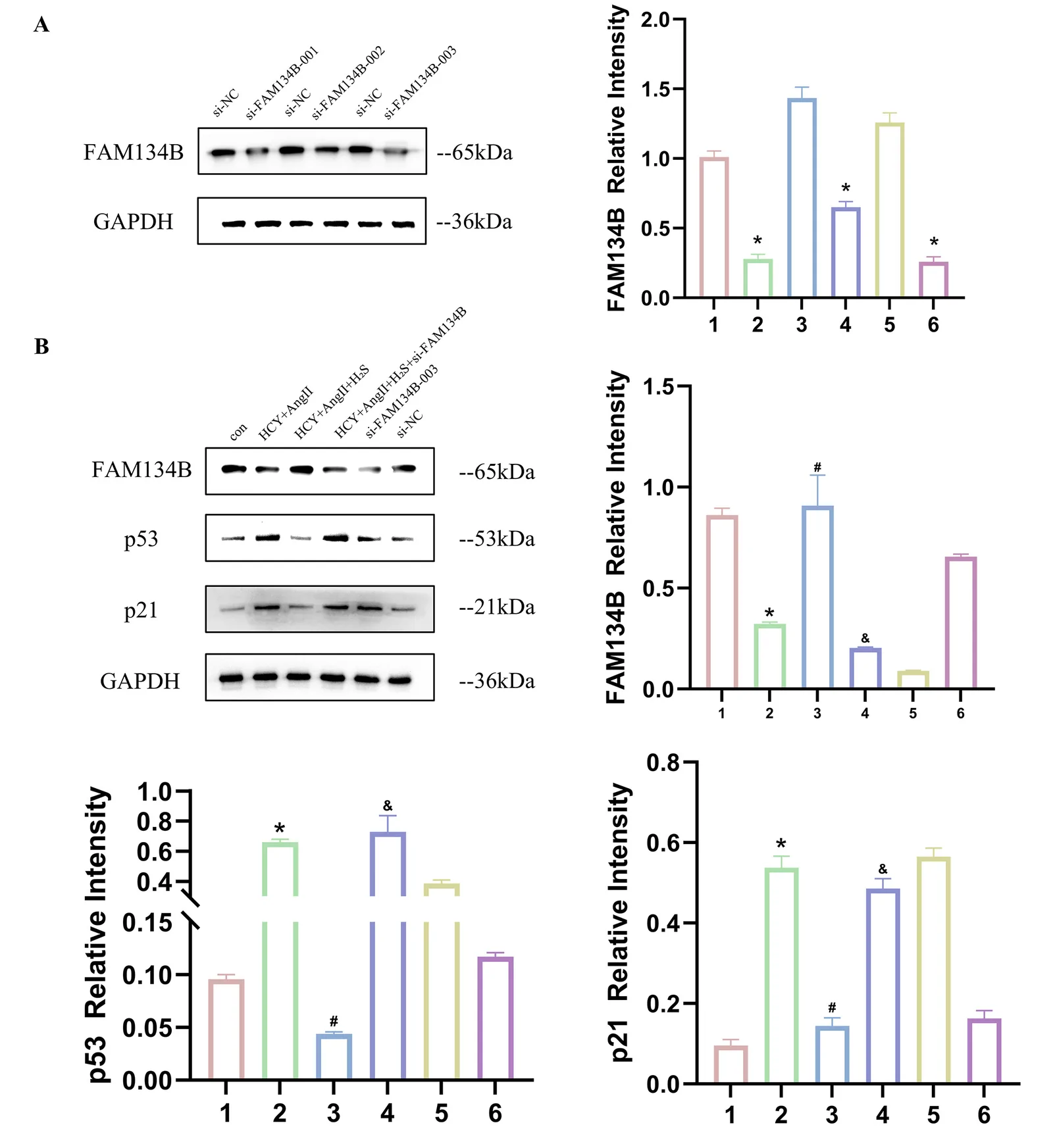
**Knockdown of FAM134B abolishes the protective effects of H_2_S against HCY+AngII-induced cardiomyocyte senescence. **(A) Screening and validation of FAM134B knockdown. Western blot analysis shows FAM134B protein levels following transfection with different siRNAs. Lane 1: si-NC; Lane 2: si-FAM134B-001; Lane 3: si-NC; Lane 4: si-FAM134B-002; Lane 5: si-NC; Lane 6: si-FAM134B-003. The graph shows quantification of FAM134B levels, confirming si-FAM134B-003 as the most efficient sequence (*p < 0.05 vs. respective si-NC controls). (B) FAM134B knockdown reverses the anti-senescent effect of H_2_S. Western blot analysis (top) and quantification (bottom) of senescence markers P21, P53, and FAM134B under different treatments. Lanes are labeled as: 1: Control; 2: HCY+AngII; 3: HCY+AngII+H_2_S; 4: HCY+AngII+H_2_S+si-FAM134B; 5: si-FAM134B; 6: si-NC. GAPDH served as a loading control. *Data in all quantitative graphs are presented as mean ± SEM from 3 independent experiments. Multiplicity-adjusted *p*-values were obtained using one-way ANOVA followed by Tukey’s multiple-comparisons test. In panels B: *p < 0.001 vs. group 1 (Control); #*p* < 0.001 vs. group 2 (HCY+AngII); &*p* < 0.001 vs. group 3 (HCY+AngII+H_2_S).

## 4. Discussion

In this study, we further investigated the molecular mechanism by which H_2_S ameliorates HCY+ AngII-induced H9c2 cardiomyocyte senescence and HH-induced myocardial fibrosis. We found that H_2_S inhibited HH-induced myocardial fibrosis by activating FAM134B-mediated ER autophagy through the PERK–eIF2α signaling pathway. We observed that hyperhomocysteine-induced hypertension triggered excessive ER stress, and H_2_S could inhibit cardiomyocyte senescence and restore ER homeostasis by inhibiting the ER stress pathway PERK–eIF2α and triggering the ER autophagy receptor FAM134B to anchor LC3. This improves myocardial fibrosis induced by hyperhomocysteine-induced hypertension. H_2_S has emerged as an important cardioprotective gasotransmitter, and H_2_S-based therapeutic strategies have attracted increasing attention in cardiovascular research [23].

### 4.1 Hyperhomocysteine-Induced Hypertension Leads to Myocardial Remodeling and Cardiomyocyte Senescence

Hypertension is a common disease that endangers human health, and it is also one of the main causes of death in modern society. Long-term pressure overload induces myocardial fibrosis, partly due to the increased concentration of AngⅡ, which promotes excessive deposition of collagen. In addition, various growth factors and cytokines stimulate fibroblasts to differentiate into myofibroblasts characterized by elevated α-smooth muscle actin (α-SMA) expression and enhanced collagen secretion, thereby exacerbating myocardial fibrosis and ultimately contributing to adverse cardiovascular events ‎[24]. At present, 75% of patients with essential hypertension in China have hyperhomocysteinemia. Hyperhomocysteinemia is directly toxic to the myocardium. Because homocysteine has a direct promoting effect on myocardial matrix metalloproteinases and myocardial fiber activity, it is more likely to cause cardiac systolic and diastolic dysfunction. Previous studies have shown that hyperhomocysteinemia can contribute to myocardial injury and cardiac remodeling [25]. This suggests that compared with simple hypertension, hypertension with hyperhomocysteinemia is more likely to result in myocardial injury and myocardial remodeling, significantly increasing mortality. Myocardial fibrosis is one of the key links in this process. The results of echocardiography, Masson staining, RT-qPCR, and WB showed that the cardiac function of HHCY group rats was significantly decreased, collagen deposition was significantly increased, and the expression of fibrosis-related proteins was significantly up-regulated, which was similar to the results of previous studies. These results suggest that hyperhomocysteine-induced hypertension can induce myocardial remodeling. However, the mechanism of myocardial remodeling is still unclear. Cardiovascular aging is associated with oxidative stress, mitochondrial dysfunction, and chronic low-grade inflammation, providing a broader context for investigating the relationship between cellular senescence and cardiac remodeling [26]. Previous studies have found that cardiomyocyte senescence can promote the occurrence and development of myocardial fibrosis, but the mechanism of cardiomyocyte senescence and its effect on myocardial fibrosis in HCY-type hypertensive diseases need to be further explored. We also found that cardiomyocyte senescence was up-regulated while inhibitory senescence was down-regulated in this model. Moreover, by treating H9c2 cells with DL-homocysteine and AngII, we found that the expression of the anti-aging protein SIRT1 was significantly reduced, suggesting that we have successfully constructed the H9c2 cardiomyocyte aging model. The increased positive rate of SA-β-Gal (SA-β-Gal) is considered to be an important marker of cellular senescence. In this study, the changes in the senescence-related protein P21 and anti-senescence-related proteins SIRT1 and SIRT2 were detected by immunofluorescence assay and Western blot. These results suggest that homocysteine-induced hypertension can induce senescence of H9c2 cells. Kwak et al. [7] confirmed that cellular senescence is involved in the development of myocardial fibrosis and its mechanism through *in vitro* and *in vivo* experiments. The present study also found that the expression of senescence-related proteins was significantly up-regulated during myocardial remodeling in hyperhomocysteinemic hypertensive rats and H9c2, which was consistent with previous results. However, the precise mechanisms linking hyperhomocysteinemia-induced hypertension to cardiomyocyte senescence and myocardial remodeling remain unclear.

### 4.2 Endoplasmic Reticulum Stress PERK–eIF2α Pathway Leads to Myocardial Cell Failure and Myocardial Fibrosis Induced by Hyperhomocysteinemia

Studies on the mechanisms of cellular senescence are often limited to oxidative stress, lipid toxicity, telomere shortening, metabolic disorders, and DNA damage. Recent studies have found that cellular senescence is closely related to cellular stress, including ER stress, oxidative stress, and mitochondrial stress. Endoplasmic reticulum (ER) stress has emerged as a critical regulator of cardiovascular remodeling and cellular senescence [27,28]. The ER is used to detect unfolded/misfolded proteins, which can lead to ER stress when there is an imbalance between their protein load and folding capacity. Excessive ER stress, with its resultant impaired protein homeostasis and chronic inflammation, as well as other accompanying cellular stresses, can significantly lead to the occurrence of cellular senescence ‎[29,30], and senescent cells also undergo ER stress with increased expression of related proteins ‎[31]. Homocysteine can induce ER stress-mediated apoptosis in cardiomyocytes, and it is believed that ER stress is a common pathway causing cardiomyocyte injury ‎[32]. The expression of ER stress proteins GRP78, CHOP, eIF2α, and caspase-12 is increased in myocardial tissues of individuals with homocysteine ‎[33]. It is speculated that endoplasmic reticulum stress may play a similar role in homocysteine-induced hypertension. In this study, we observed that the expression of ER stress proteins BIP and CHOP, PERK, and eIF2α was significantly upregulated in homocysteine-induced hypertensive rats, and the expression of ER stress proteins was increased in H9c2 cells in the senescent state. This suggests that the ER stress PERK–eIF2α pathway inhibits cardiomyocyte senescence and is associated with myocardial fibrosis induced by hyperhomocysteine-induced hypertension. This has not been studied previously, but it is consistent with the effect of ER stress on cellular senescence in type 2 diabetes and other endocrine diseases ‎[34]. It has been previously confirmed that ER autophagy can inhibit excessive ER stress by inducing the autophagy response, and then reassembling degraded ER fragments into new ERS, thereby restoring the normal state of the ER. Therefore, ER autophagy seems to be an important method to restore ER homeostasis and has a cytoprotective effect ‎[35]. These results suggest that ER stress is enhanced in hyperhomocysteine-induced hypertension, and enhancing ER autophagy and attenuating stress to inhibit cellular senescence may block myocardial remodeling in this disease.

### 4.3 FAM134B-Mediated Endoplasmic Reticulum Autophagy Inhibits Cardiomyocyte Senescence and Myocardial Fibrosis Induced by Hyperhomocysteine-Induced Hypertension

ER autophagy limits ER stress and maintains ER homeostasis, thereby potentially inhibiting cell senescence. Previous studies have shown that FAM134B is an essential factor for mediating ER phagocytosis, and it has an LIR at its carboxyl terminus in the cytoplasm, both of which mediate the process of ER autophagy ‎[11,36]. Recent studies have further confirmed that FAM134B-mediated ER-phagy plays a crucial role in maintaining ER homeostasis and regulating cellular stress responses [37]. FAM134B-mediated autophagy in the ER attenuates advanced glycation end product-induced senescence in human nucleus pulposus cells ‎[38]. There are few studies on FAM134B-mediated endoplasmic reticulum autophagy and cardiomyocyte aging. FAM134B and LC3-II/I expression are increased in septic mice and lipopolysaccharide-treated cardiomyocytes, and FAM134B-mediated ER autophagy has a protective effect against myocardial injury by reducing inflammation and tissue apoptosis ‎[39]. More recently, Li et al. [40] demonstrated that activation of FAM134B-mediated ER-phagy alleviated myocardial ischemia-reperfusion injury by promoting autophagic flux and suppressing ER stress, whereas FAM134B knockdown partially reversed these protective effects. We sought to investigate whether FAM134B-mediated autophagy is involved in cardiomyocyte senescence induced by homocysteine-induced hypertension. In this study, in H9c2 cells treated with DL-homocysteine and AngII, endoplasmic reticulum autophagy was significantly attenuated compared with the control group by electron microscopy. Compared with the control group, the expression of FAM134B and LC3B, the key factors of endoplasmic reticulum autophagy, was decreased in both H9c2 cells and rats with myocardial fibrosis. These results indicate that endoplasmic reticulum autophagy is decreased during cardiomyocyte senescence and myocardial remodeling induced by homocysteine-induced hypertension. The expression of cellular senescence proteins P53 and P21 was significantly increased after transfection of siRNA-FAM134B using siRNA silencing technology, which further suggested that FAM134B-mediated endoplasmic reticulum autophagy is essential in inhibiting senescence in H9c2 cells. This finding is consistent with previous reports that ER autophagy has a protective effect on aging in other cells and cardiomyocytes. Recent evidence also indicates that enhanced BiP-dependent ER-phagy involving the BiP–FAM134B interaction protects against pressure overload-induced cardiac hypertrophy, fibrosis, and heart failure [41]. These results suggest that the decreased endoplasmic reticulum autophagy may be one of the key mechanisms of cell senescence and myocardial remodeling induced by homocysteine-induced hypertension. However, although our findings indicate a strong association between ER-phagy and cardiomyocyte senescence, the causal relationship requires further validation.

### 4.4 H_2_S Can Improve Myocardial Fibrosis in Hyperhomocysteine-Induced Hypertension by Inhibiting PERK–eIF2α and Promoting FAM134B-Mediated Endoplasmic Reticulum Autophagy to Inhibit Cell Senescence

H_2_S is an endogenous gas signaling molecule found to be widely distributed in various parts of the body, with recognized anti-inflammatory and cytoprotective effects [42,43]. H_2_S exerts anti-inflammatory and anti-fibrotic effects, contributing to its protective role in cardiovascular diseases [44,45,46,47]. Extensive studies have shown its potential protective effects against diabetic heart disease, ischemia/reperfusion injury, myocardial infarction, and drug cardiotoxicity ‎[48,49]. Previous studies found that H_2_S played a protective role in animal models such as diabetic cardiomyopathy‎ and dilated cardiomyopathy. In addition, a novel mitochondrial-targeted H_2_S donor, AP39, has been found to improve myocardial fibrosis induced by high homocysteine and myocardial infarction through mitophagy ‎[50]. Exogenous H_2_S has also been shown to reduce blood pressure in spontaneously hypertensive rats and inhibit collagen synthesis in cardiac fibroblasts through the TGF-β/Smad signaling pathway. It should be noted that the biological effects of H_2_S are dose-dependent, and excessive concentrations may exert cytotoxic effects, emphasizing the importance of dose optimization in therapeutic applications [51,52,53]. It reduces collagen content in the myocardium and subsequently improves collagen deposition in the myocardium of spontaneously hypertensive rats and alleviates myocardial remodeling ‎[54]. These studies all suggest that H_2_S improves myocardial fibrosis. In this study, it was found that H_2_S intervention reduced myocardial collagen content, myocardial fibrin, Collagen I and Collagen III, decreased the expression of senescence proteins P21 and P16, increased the expression of anti-senescence proteins SIRT1 and Sirt2, and improved the related indicators of cardiac function. H_2_S was also found to improve cardiomyocyte senescence in the H9c2 cardiomyocyte senescence model. These results suggest that H_2_S can improve myocardial fibrosis and cellular senescence induced by hyperhomocysteinemia and has a protective effect on cardiac function.

These results suggest that PERK–eIF2α-mediated ER stress and FAM134B-mediated ER autophagy are related to cell senescence and myocardial fibrosis in homocysteine-induced hypertension. We used H_2_S intervention in rats and cells to explore how H_2_S exerts its cardioprotective effect. We found that the endoplasmic reticulum stress-related proteins BIP, CHOP, PERK, and eIF2α were significantly down-regulated after treatment with H_2_S, but the effect of H_2_S on cell senescence was significantly reduced after treatment with a PERK agonist. This suggests that H_2_S may inhibit cardiomyocyte senescence through the PERK–eIF2α pathway. This is consistent with previous studies showing that H_2_S can reduce the expression of ER stress proteins in hyperhomocysteinemia myocardial tissue, thereby exerting a cardioprotective effect [25,33]. Using immunofluorescence, cell interference, WB, electron microscopy, and other techniques, we observed that H_2_S could significantly increase the expression of ER autophagy-related proteins FAM134B and LC3A/B, increase the colocalization of FAM134B and LC3B, and increase the occurrence of ER autophagy, and that this process could be blocked by siRNA-FAM134B. This suggests that H_2_S can inhibit cellular senescence and ultimately improve myocardial remodeling by regulating FAM134B-mediated ER autophagy. These studies confirmed that the ER stress pathway PERK–eIF2α is enhanced in cardiomyocytes, while FAM134B-mediated ER autophagy is attenuated in hyperhomocysteinemic hypertension. H_2_S can inhibit cell senescence and improve fibrosis through these mechanisms. To further explore the relationship between H_2_S and the ER stress pathway PERK–eIF2α and FAM134B-mediated autophagy, the ER autophagy proteins FAM134B and LC3A/B were significantly down-regulated after treatment with the PERK pathway agonist in H_2_S-treated groups. This suggests that FAM134B-mediated autophagy can be up-regulated by inhibiting the PERK–eIF2α pathway, thereby playing a protective role. These findings indicate that the cardioprotective effects of H_2_S are mediated through multiple interconnected pathways, including regulation of oxidative stress, mitochondrial function, and cellular signaling networks [55].

## 5. Limitations

Several limitations of the present study should be acknowledged. First, H9c2 cells were used as an *in vitro* cardiomyocyte model, which may not fully reflect the characteristics of primary cardiomyocytes *in vivo*. Second, although alterations in FAM134B expression, LC3B levels, immunofluorescence colocalization, and electron microscopy findings suggested changes in ER-phagy activity, ER-phagy flux was not directly quantified. Third, the mechanistic conclusions were mainly based on protein expression analyses, pharmacological intervention, and siRNA knockdown experiments, and additional rescue experiments are still required to further establish the causal relationship between PERK–eIF2α signaling, FAM134B-mediated ER-phagy, and the cardioprotective effects of H_2_S. Therefore, future studies using primary cardiomyocytes and more comprehensive functional validation approaches are warranted.

## 6. Conclusions

In this study, we found that H_2_S could promote FAM134B-mediated autophagy through PERK–eIF2α to inhibit cell senescence and improve myocardial remodeling induced by hyperhomocysteinemia, suggesting that ER autophagy may be an important part of the process of myocardial cell senescence. H_2_S can upregulate FAM134B-mediated endoplasmic reticulum autophagy to inhibit cardiomyocyte senescence. These findings suggest that modulation of the PERK–eIF2α/FAM134B-mediated ER-phagy pathway may represent a potential strategy for attenuating myocardial remodeling induced by hyperhomocysteinemia.

## Data Availability

The datasets used and analyzed during the current study are available from the corresponding author on reasonable request.
