# Supplementary material for: Exogenous Hydrogen Sulfide Promotes FAM134B-Mediated Endoplasmic Reticulum Autophagy and Inhibits Cell Aging by PERK–eIF2α and Improves Homocysteinemic Hypertensive-Induced Myocardial Fibrosis
Source: Rev Cardiovasc Med. 2026 Oct 14;27(10):51893. doi: 10.31083/RCM51893 (PMC13732668; doi:10.31083/RCM51893)
Supplement: Supplementary file 1 [file 2153-8174-27-10-51893-s1.zip › ARRIVE_Checklist.pdf]

NOTE: Please save this file locally before filling in the table, DO NOT work on the file within your internet browser as changes will not be saved. Adobe Acrobat Reader (available free here) is recommended for completion.

The ARRIVE guidelines 2.0: author checklist

The ARRIVE Essential 10

These items are the basic minimum to include in a manuscript. Without this information, readers and reviewers cannot assess the reliability of the findings.

Item		Recommendation	Section/line number, or reason for not reporting
**Study design**	1	For each experiment, provide brief details of study design including:	
		a. The groups being compared, including control groups. If no control group has been used, the rationale should be stated.	Sec. 2.6: four animal groups (Model, Model+H2S, Control, Control+H2S). Animal results: Secs. 3.1-3.3, 3.5-3.6.
		b. The experimental unit (e.g. a single animal, litter, or cage of animals).	Sec. 2.6; the individual rat is the apparent experimental unit, but this is not explicitly stated.
**Sample size**	2	a. Specify the exact number of experimental units allocated to each group, and the total number in each experiment. Also indicate the total number of animals used.	Sec. 2.6: total 30 animals; n=8,8,7,7. Table 1 reports n=7/group (discrepancy).
		b. Explain how the sample size was decided. Provide details of any *a priori* sample size calculation, if done.	Not reported: sample-size rationale or a priori calculation.
**Inclusion and exclusion criteria**	3	a. Describe any criteria used for including and excluding animals (or experimental units) during the experiment, and data points during the analysis. Specify if these criteria were established *a priori*. If no criteria were set, state this explicitly.	Not reported: prespecified inclusion/exclusion criteria.
		b. For each experimental group, report any animals, experimental units or data points not included in the analysis and explain why. If there were no exclusions, state so.	Not reported. Methods allocate n=8,8,7,7, but Table 1 reports n=7/group; attrition/exclusion is unexplained.
		c. For each analysis, report the exact value of *n* in each experimental group.	Partial: Table 1 n=7/group; Fig. 1 n=7-8; Fig. 2 reports fields/experiments. Exact animal n is not stated for each analysis.
**Randomisation**	4	a. State whether randomisation was used to allocate experimental units to control and treatment groups. If done, provide the method used to generate the randomisation sequence.	Sec. 2.6: random allocation stated; random-sequence generation method not reported.
		b. Describe the strategy used to minimise potential confounders such as the order of treatments and measurements, or animal/cage location. If confounders were not controlled, state this explicitly.	Not reported: control of treatment/measurement order, cage location, or other potential confounders.
**Blinding**	5	Describe who was aware of the group allocation at the different stages of the experiment (during the allocation, the conduct of the experiment, the outcome assessment, and the data analysis).	Not reported: blinding during allocation, conduct, outcome assessment, or data analysis.
**Outcome measures**	6	a. Clearly define all outcome measures assessed (e.g. cell death, molecular markers, or behavioural changes).	Animal outcomes: Secs. 3.1-3.3, 3.5-3.6 (BP/Hcy, cardiac function, fibrosis, PERK, FAM134B/LC3); methods: Secs. 2.5-2.9.
		b. For hypothesis-testing studies, specify the primary outcome measure, i.e. the outcome measure that was used to determine the sample size.	Not reported: primary outcome used for sample-size determination.
**Statistical methods**	7	a. Provide details of the statistical methods used for each analysis, including software used.	Sec. 2.10: mean +/- SEM; one-way ANOVA + Dunnett's post hoc test; GraphPad Prism; p<0.05.
		b. Describe any methods used to assess whether the data met the assumptions of the statistical approach, and what was done if the assumptions were not met.	Not reported: checks of statistical assumptions or alternative methods.
**Experimental animals**	8	a. Provide species-appropriate details of the animals used, including species, strain and substrain, sex, age or developmental stage, and, if relevant, weight.	Sec. 2.6: male SHR (n=16) and adult male WKY (n=14), rats, 200 +/- 20 g; exact age not reported.
		b. Provide further relevant information on the provenance of animals, health/immune status, genetic modification status, genotype, and any previous procedures.	Sec. 2.6: supplier and strain/background stated; substrain, health/immune status, genotype and prior procedures not reported.
**Experimental procedures**	9	For each experimental group, including controls, describe the procedures in enough detail to allow others to replicate them, including:	Secs. 2.5-2.9, esp. 2.6: methionine model, NaHS/saline treatment, anesthesia/euthanasia, and tissue assays.
		a. What was done, how it was done and what was used.	
		b. When and how often.	Sec. 2.6: 1-wk acclimatisation; methionine for 10 wk; NaHS/saline daily for 4 wk.
		c. Where (including detail of any acclimatisation periods).	Sec. 2.6: 24 +/- 3 C; 12-h light/dark; food/water ad libitum; 1-wk acclimatisation.
		d. Why (provide rationale for procedures).	Sec. 2.6: rationale for SHR/WKY pairing/model stated; rationale for NaHS dose/route/duration not reported.
**Results**	10	For each experiment conducted, including independent replications, report:	
		a. Summary/descriptive statistics for each experimental group, with a measure of variability where applicable (e.g. mean and SD, or median and range).	Animal results: Secs. 3.1-3.3 and animal portions of 3.5-3.6; Table 1; Figs. 1, 2 and 4. Data generally mean +/- SEM.
		b. If applicable, the effect size with a confidence interval.	Not reported: effect sizes with confidence intervals.
